# Ameloblastoma during pregnancy: a case report

**DOI:** 10.1186/s13256-016-1025-1

**Published:** 2016-09-06

**Authors:** Helbert Eustáquio Cardoso da Silva, Erika do Socorro Ramos Costa, Antônio Carlos Quintão Medeiros, Paulo Sérgio dos Santos Pereira

**Affiliations:** 1 Post-Graduation Program, Foundation for Teaching and Research in Health Sciences, Brasilia, Brazil; 2 Department of Dentistry, Paulista University, Brasília Campus, Brasilia, Brazil; 3 Dentistry Care, Sobradinho Regional Hospital, Sobradinho, Brasilia, Brazil; 4 Condomínio Mansões Entrelagos Etapa 1 conjunto S casa 9, Paranoá, Brasília, CEP: 73255-900 DF Brazil

**Keywords:** Ameloblastoma, Pregnancy, Odontogenic tumors

## Abstract

**Background:**

Ameloblastomas are rarely found in pregnant women, with only two cases reported in the scientific literature. We report the first case of ameloblastoma in a pregnant woman in Brazil.

**Case presentation:**

A 27-year-old white woman, 12-weeks pregnant, presented with a large mass in her right posterior mandible. Panoramic radiography revealed a lesion involving her mandibular right first molar with displacement of her mandibular right third molar and impairment of the mandibular bone base. The results of an incisional biopsy led to a diagnosis of acanthomatous ameloblastoma. We fixed Erich arch bars to both dental arches and performed an en-bloc resection surgery under general anesthesia for tumor removal. She was then treated by maxillomandibular rigid fixation with the installation of a 2.7 mm non-locking reconstruction plate. So far, she has presented no motor deficits, chewing difficulties, or relevant asymmetries. The tumor showed no recurrence after the first year (pregnancy period) and post-surgery radiographic follow-up revealed a reduction in the surgical area after osseous growth in the margins of the lesion. Although she displayed no systemic comorbidities that affected pregnancy, the fetus was born with alobar holoprosencephaly.

**Conclusions:**

The possible influence of pregnancy hormones on the growth and development of tumors in general and ameloblastoma in particular, is still not explained in the literature. However, evidence reveals that the issue should be further studied. Although en-bloc resection surgery is considered a radical method of treatment, it is an effective alternative in ameloblastoma removal, presenting low rates of recurrence.

## Background

Ameloblastoma is an aggressive infiltrating odontogenic tumor with high recurrence rates. It represents 11 % of all odontogenic tumors and less than 1 % of all tumors affecting the jaws, with a rare ability to metastasize [1]. It is an asymptomatic slow-growing tumor characterized by cortical bone expansion or perforation and infiltration to soft tissues [2]. The disease commonly appears in the third to seventh decades of life, with no gender preference [3]. It mainly occurs in the mandibular bone (85 % prevalence) with predilection for the posterior region of the molars, on the ascending branch of the mandible. Less frequent cases have been reported in the premolar and anterior regions [4].

Ameloblastoma radiology usually presents a unilocular or multilocular radiolucency; the latter has a soap bubble appearance, indicating that it might be divided into several bone spaces by trabeculae. Ameloblastomas are often associated with the presence of unerupted teeth. The teeth associated with ameloblastoma are vital, presenting in some cases with migration, mobility, and root resorption [3].

Surgical treatment for this type of tumor extends from conservative forms, such as curettage, enucleation, and cryosurgery, to more radical forms, such as marginal resection, en-bloc resection, or segmental/hemiresection [5]. Recurrence rates vary according to the type of lesion and surgical modality, ranging from 7 to 25 % [6, 7] after radical surgery and 20 [7] to 33 % [4] for conservative techniques.

Ameloblastoma cases during pregnancy are still scarce in the literature. Here we present a rare clinical case of ameloblastoma in a 27-year-old white pregnant woman, the first case reported in the Brazilian population.

## Case presentation

A descriptive case study conducted at Hospital Regional de Sobradinho (HRS) after Federal District health ethics committee approval (CEP/SESDF) on 3 August 2015, CAAE - 47521015.6.0000.5553, number: 1,167,855.

These data were recorded in the free informed consent form signed by the patient. The case was selected due to the rarity of ameloblastoma occurrence in pregnant women.

A 27-year-old white woman, 12-weeks pregnant, presented with increased mandibular volume in the right posterior region of her mandible. She had been complaining of pain and bleeding for months, feeling uncomfortable on her face when sleeping on her right side. She described initial observation of the pain in the middle of the second month of pregnancy. The pain increased in the third month, which led her to search for public dental care. She was observed until week 22 because surgery was recommended only after the first trimester of pregnancy.

According to hospital records, she was a non-cigarette smoking and non-alcoholic primipara reporting no relevant systemic comorbidities. She had attended 16 prenatal appointments due to the diagnosis of alobar holoprosencephaly at week 30 of pregnancy. She reported no pregnancy complications and exhibited normal values in blood tests.

An intraoral examination showed mandibular/buccal expansion in the right alveolar process, close to her molars, with deviation of her right mandibular third molar. Mucosa was ulcerated and bleeding due to trauma caused by the maxillary teeth cusps, which was the source of the pain. She had had the right mandibular second molar extracted on an unknown date due to extensive tooth decay and pain. Imaging examination of the lesion region dating back to 2005 revealed no abnormalities from the surgery.

**Fig. 1:**
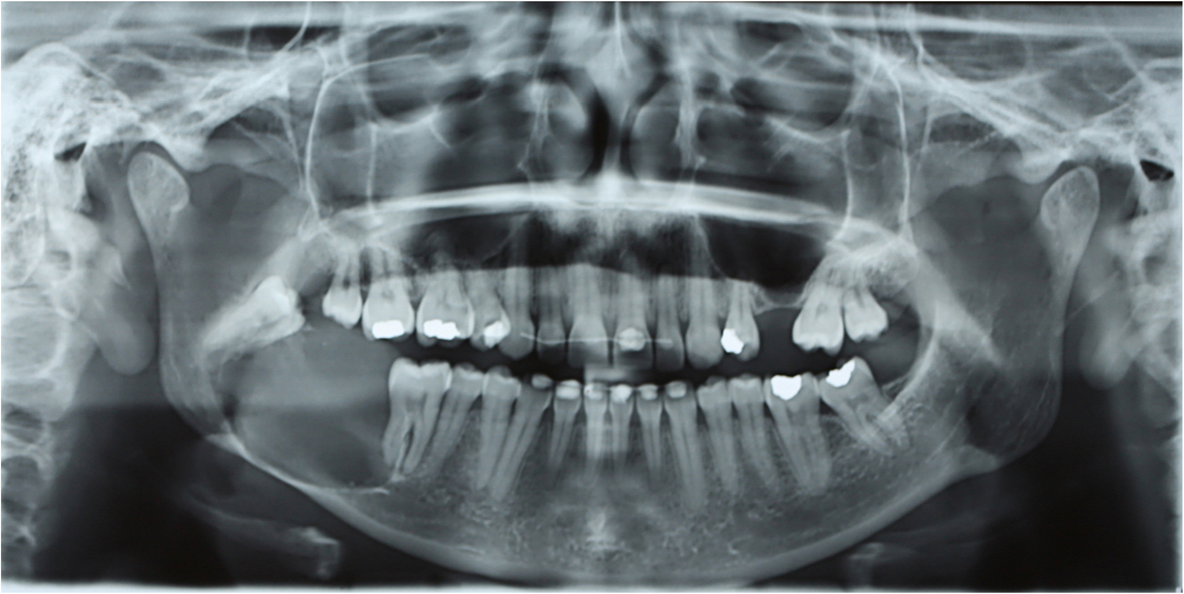
Initial radiography with radiolucent image located at the right side of the ascending branch of the mandible

A panoramic X-ray showed a large unilocular osteolytic lesion extending from her right mandibular first molar to the ascending ramus. Images also showed displacement of her right mandibular third molar and involvement of the mandibular bone base (Fig. 1).

**Fig. 2:**
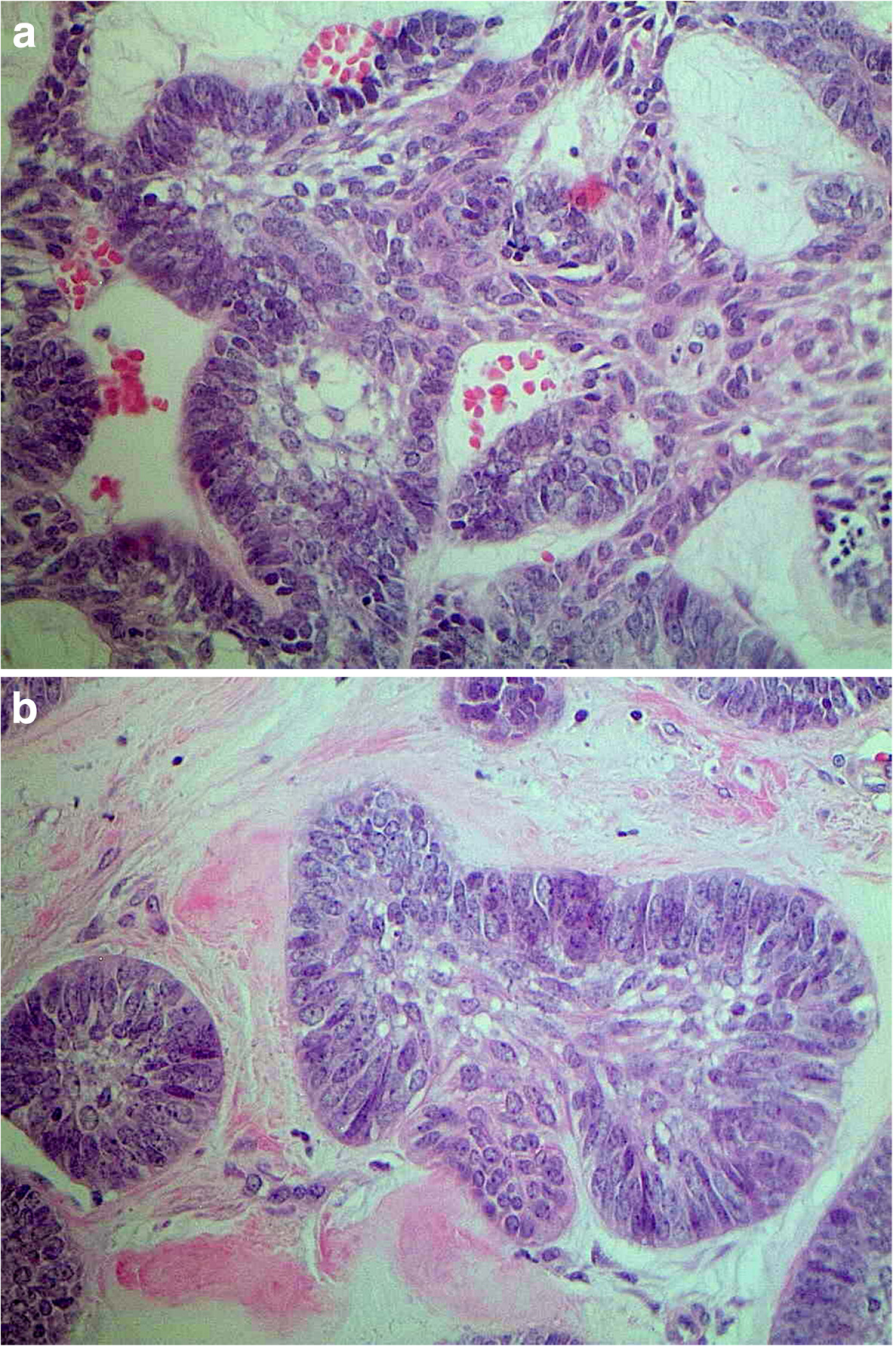
**a** Acanthomatous ameloblastoma: cords and network of odontogenic epithelium arranged in palisade exhibiting reversed polarity cores (×400). **b** Acanthomatous ameloblastoma: cells with squamous differentiation can be observed in the center (×400)

An incisional biopsy revealed acanthomatous ameloblastoma with nests of cords and islands of basaloid odontogenic palisading epithelial cells. The columnar cells showed elongated nuclei with inconspicuous nucleoli and exhibited inverted polarization opposite to the basement membrane (Fig. 2a). The central area of the islands consisted of loosely arranged cells resembling stellate reticulum. These cells may exhibit squamous differentiation, characterizing the acanthomatous histological variant (Fig. 2b).

She was admitted to our surgical center to be submitted to en-bloc resection surgery and fixation of a reconstructive non-locking plate under general anesthesia; during surgery she was in a supine position, and she was monitored and pre-oxygenated. Anesthesia was induced by rapid sequence of Sellick’s maneuver with fentanyl 200 mg, propofol 150 mg, Quelicin (succinylcholine) 60 mg and nasal intubation. Maintenance was performed with oxygen, nitrous oxide, and sevoflurane.

Surgical access was provided with Erich arch bar applications in both dental arches and through intramuscular incision in the right cervical region. Prior to tumor removal, we adapted a 2.7 mm reconstructive non-locking plate by boring holes in it. The plate was removed and the maxillomandibular fixation was released for intrasulcular incisions and gingiva detachment.

Osteotomy was performed from the mesial of her second premolar to half of the ascending ramus. Maxillomandibular fixation was redone and the 2.7 mm reconstructive non-locking plate was re-installed. Extraoral suturing, fixation release and intraoral suturing were performed in sequence. At the end of the surgery, we used elastics for occlusal maintenance.

**Fig. 3:**
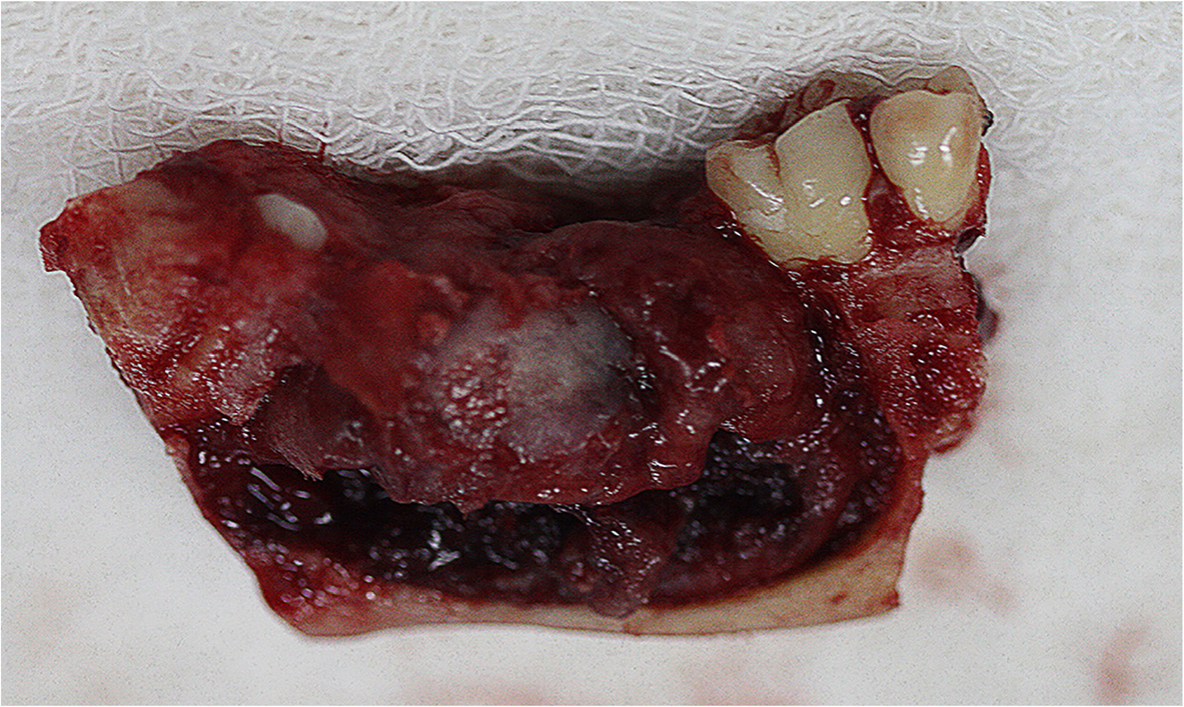
Fragment of the mandible removed after surgery and submitted to confirm the initial biopsy

We submitted the surgical specimen to a new histopathological analysis, which confirmed the previous diagnosis of ameloblastoma (Fig. 3).

**Fig. 4:**
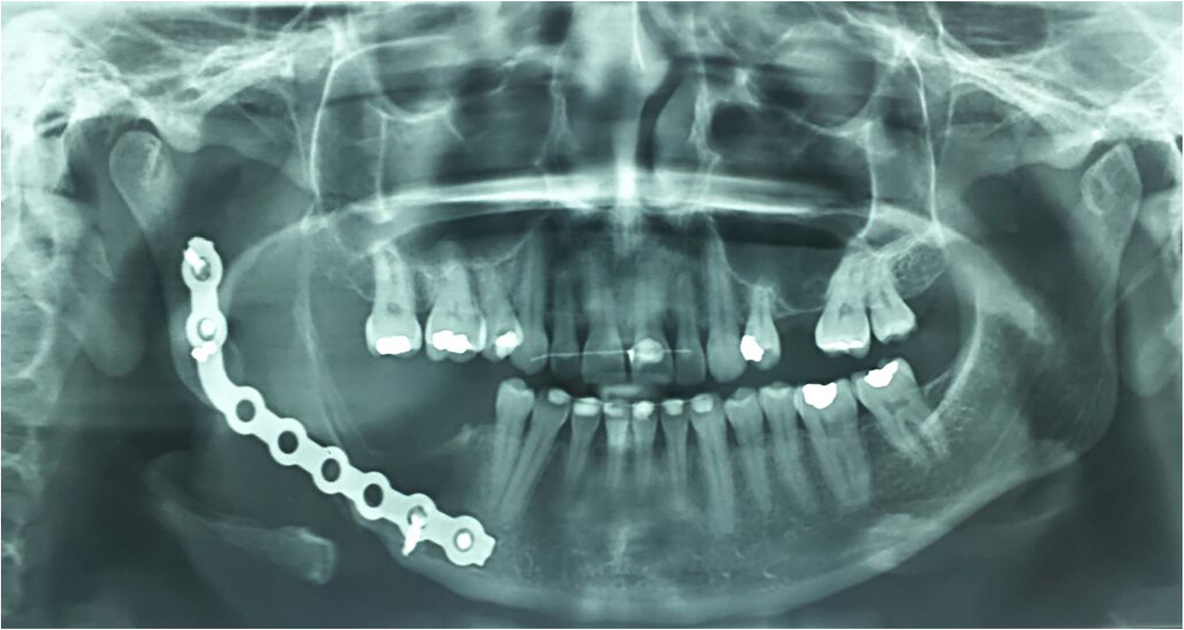
Panoramic radiography at 24-month follow-up

She was then monitored for 24 months at the Sobradinho Regional Hospital dental care unit, having evolved well without any signs of recurrence. She presented no motor deficits, chewing difficulties, or relevant asymmetries. She even reported improvement in chin skin sensitivity. The tumor showed no recurrence after the first year (pregnancy period) and post-surgery radiographic follow-up revealed a reduction of surgical area after osseous growth in the margins of the lesion. In the second year, radiography revealed a suggestive image formation and osseous growth without tumor recurrence (Fig. 4). Due to our patient’s desire to become pregnant again, she decided to wait for a second reconstructive stage.

## Discussion

The World Health Organization classifies ameloblastoma into three subtypes of odontogenic tumor: multicystic, unicystic and peripheral [8]. Ameloblastoma is an asymptomatic locally invasive slow-growing tumor (benign) characterized by cortical bone expansion or cortical perforation and infiltration to soft tissue. It is regarded as a potentially malignant tumor. We reported a case of unicystic subtype ameloblastoma. Unicystic ameloblastomas affect younger patients, between the second and third decades of life, and are often associated with impacted teeth, especially the second and third molars [9, 10].

The clinical features most frequently associated with unicystic ameloblastoma include swelling of the mandibular region and/or absence of a tooth from its place in the arch in the region of the tumor [11]. Crepitation may occur depending on tumor volume. Aspiration yields a yellow liquid, similar to odontogenic cysts [12]. Cysts are generally painless and hardly perceived by the patient in early stages. Due to its slow growth, the tumor is generally tentatively diagnosed through radiographic examination [13, 14].

Some histological variants of ameloblastoma include follicular, plexiform, granular cell, acanthomatous, basal cell, and desmoplastic types [15]. Acanthomatous ameloblastoma is a rare variant of the disease exhibiting solid epithelial cell nests with peripheral palisading ameloblastic cells and central squamous cell differentiation. Due to similar patterns, acanthomatous ameloblastoma may be confused with a squamous cell carcinoma and may also appear as a “hybrid” lesion of ameloblastoma admixed with a pronounced desmoplastic pattern [16].

Ameloblastoma in the presented case follows the pattern of other cases described in the literature. What makes this case report relevant is the development of ameloblastoma at week 12 of pregnancy. This fact suggests that hormones released during pregnancy are probably able to influence growth and development of odontogenic tumors.

Because she was in her first trimester of pregnancy when she came to our hospital dental service, computed tomography (CT) was not recommended. A panoramic radiography was done instead because a cone beam computed tomography (CBCT) examination, which could be used as a supplementary examination option, is not available in the public health system. The use of panoramic radiography represents a tenfold reduction in the amount of radiation absorbed by the patient compared to periapical radiography. For pregnant women, since the intrauterine dose absorbed is less than 10^-6^ absorbed radiation dose (rad), the risk of mental abnormalities due to dental radiology is considered nonexistent [17].

Fetal alobar holoprosencephaly, diagnosed after ameloblastoma removal, shows no connection with the surgery. The condition is linked to chromosomal changes such as Patau syndrome (trisomy 13) and Edwards syndrome (trisomy 18) [18]. Some studies also established a relationship between diabetes and holoprosencephaly [19]. Alobar holoprosencephaly accompanied by cebocephaly is characterized by absent nose or single-nostril flattened nose and closely set eyes. In the present case, the baby died of pneumonia 10 months later.

Studies show conflicting results on the development of odontogenic tumors during pregnancy. Sekiya *et al*. reported a case of adenomatoid odontogenic tumor (AOT) in which estrogen receptors (located in tumor cell nuclei) were positive for B-cell lymphoma 2 (Bcl-2), suggesting that the survival of tumor cells was facilitated by Bcl-2 upon continuous stimulation of estrogen during pregnancy [20]. However, another study on AOT during a pregnancy study by Bhandari and Kothan revealed AOT with a cystic component with no dependence on estrogen or progesterone for its growth [21].

Only two cases of ameloblastoma tumors during pregnancy have been reported in the literature [22, 23]. Herberts and Sandstrom [22] showed that pregnancy hormones may influence the growth and development of tumors. Estrogen and gonadotropin (considered etiological agents of tumors), for example, can induce hyperplasia because of their high concentration levels during pregnancy.

Another study reported by Gordy et al. [23] suggested that hormonal action modulates the lesion during pregnancy, promoting rapid growth of the ameloblastoma. Pregnancy hormones promote development, growth, and birth of the newborn. These hormones may stimulate the production of several substances that, alone or in combination with each other, encourage maternal benign or malignant tumor lesion growth, although no confirmatory evidence of that has yet been presented.

In the cases reported in the literature, the women were 7-weeks [23] and 36-weeks [22] pregnant. Our patient was in week 12 of her pregnancy. Apparently, there is no specific period for the development of ameloblastoma during pregnancy. As for the demographic details, one study reports the case of an African-American patient [23] and another case does not mention the ethnicity of the patient [22]. Our patient is white. Based on the available data, we can assume that the disease has no racial preference, although no cases of Asian patients have been reported. Lesions were located in the jaw in both studies [23, 24], showing lingual expansion of the lower molars [24] and expansion of buccal surface [23] in the second premolar lower right region. This feature can represent a preference for the mandibular posterior region, similar to our findings. In both studies [23, 24], erythema was not associated with the lesion and increased mucous or swollen jaw were not identified. However, in one case [24], the lesion was palpable, but presented no fluctuation in its 5-year evolution period. In the other case [23], the lesion was soft and fluctuating due to extraction of the right mandibular second premolar 3 years before. This difference can be explained by the evolution time of the lesions before pregnancy, with or without cystic degeneration.

Both studies [22, 23] considered conservative surgery as the first surgical option before performing en-bloc resection. However, the physicians modified their treatment plan, performing en-bloc resection initially due to tumor growth, because there had been delay on the part of the patient while she contemplated termination of pregnancy [23], and lesion recurrence [22]. This emphasizes the need for the strict monitoring of patients in order to prevent tumor complications due to a lack of treatment during pregnancy.

In the case of not treating the ameloblastoma, the patient may run the risk of a pathologic fracture of the lower jaw base [24], large lesions [24], difficulties in occlusion, or recurrent trauma due to biting the lesion, in addition to the displacement of structures such as the tongue, floor of the mouth, or teeth, with the growth of the lesion. However, unicystic ameloblastoma is less aggressive with a better prognosis and reduced recurrence rates in comparison to multicystic ameloblastoma, which is locally invasive and highly destructive, because it tends to infiltrate cancellous bone [25].

Acanthomatous-type ameloblastomas have a recurrence rate of 16.2 %. Resection of unicystic ameloblastoma is described to have the lowest recurrence rate (3.6 %) if bone margins are removed appropriately. A conservative method of treatment resulted in an 18 % recurrence rate. The aim of the conservative method is to reduce the size of unicystic ameloblastoma; it is not a popular method, but it is more beneficial for severely ill patients or those with a huge lesion [11].

Regarding surgical risk, due to the fact that a unicystic ameloblastoma has a lower recurrence rate and is less aggressive, removal can be delayed. However, the possibility of pathologic fracture of the jaw and increased tumor volume were the main reasons we performed the surgical procedure after our patient completed the first trimester of pregnancy, when the maturation of the vital structures of her fetus had already been completed. Sevoflurane has excellent safety ratings, although there are rare single case reports of severe acute liver injury similar to halothane hepatitis, and it is the preferred agent because it reduces mucous membrane irritation [26]. Studies with animals failed to demonstrate risk to the fetus and there are no controlled studies in pregnant women [27].

## Conclusions

The possible influence of pregnancy hormones on the growth and development of tumors, particularly ameloblastoma, is not explained in the literature. However, evidence suggests that the issue should be better explored by further studies. En-bloc resection surgery, although a radical method of treatment, is an effective alternative for ameloblastoma removal, presenting lower recurrence rates.

### Patient’s perspective

I write the following to provide assistance to the case report written about my disease and care. I have no medical or odontological knowledge, so I only write from my own perspective and experience.

I sought the dental service that serves my community because I started to feel uncomfortable on my face when I slept on the right side. I was in the third month of pregnancy and, fearing a toothache during that period, I looked for a dentist. The dentist examined my mouth and noticed something different on my face. One side was slightly higher than the other. She asked my doctor’s opinion about having a CT scan to identify the reason for the growth. The doctor, however, said that since I was pregnant, a CT scan was not suitable because of the radiation. An X-ray was taken instead.

After the results of the examination-ray, the dentist told me I had an injury on the bone of the right mandible and referred me to the center for dental specialties at Sobradinho Regional Hospital for a consultation with a specialist. The specialist dentist said it was an injury involving the right side of my jaw and that surgery would be necessary to remove it. He explained that the lesion was large and it would be removed with a part of the injured bone and teeth involved. After removal, he would use a metal bar to join the ends. I was worried about the procedure because I thought it could affect my baby somehow. The dentist explained the risks of not removing the lesion and told me what the best time during pregnancy to do the procedure was. Pre-surgical tests were done to check my health and I obtained authorization from my prenatal doctor for the surgery.

The surgery was performed at the hospital’s surgical center on 17 March 2014. I stayed in the hospital for a day for observation and my bite was restrained for rapid healing. The greatest difficulty after surgery was the inability to eat solid food in the first few weeks. Another thing that bothered me was sleeping because of the post-surgical pain. After the surgery, I was accompanied by the professionals who treated me and had no problems during pregnancy. I go to the dentist regularly for routine screening to see if everything is still okay. The scar was along the skin folds of my neck, so it does not appear on my face.

## Abbreviations

AOT: Adenomatoid odontogenic tumor
Bcl-2: B-cell lymphoma 2
CBCT: Cone beam computed tomography
CT: Computed tomography
HRS: Hospital Regional de Sobradinho
rad: Absorbed radiation dose.
